# Unusual spine anatomy contributing to wrong level spine surgery: a case report and recommendations for decreasing the risk of preventable 'never events'

**DOI:** 10.1186/1754-9493-5-33

**Published:** 2011-12-14

**Authors:** Emily M Lindley, Sergiu Botolin, Evalina L Burger, Vikas V Patel

**Affiliations:** 1 Department of Orthopaedics, University of Colorado Denver, Denver CO, USA

**Keywords:** Wrong site surgery, wrong level spine surgery, cervical ribs, abnormal segmentation, never event

## Abstract

**Background:**

Wrong site surgery is one of five surgical "Never Events," which include performing surgery on the incorrect side or incorrect site, performing the wrong procedure, performing surgery on the wrong patient, unintended retention of a foreign object in a patient, and intraoperative/immediate postoperative death in an ASA Class I patient. In the spine, wrong site surgery occurs when a procedure is performed on an unintended vertebral level. Despite the efforts of national safety protocols, literature suggests that the risk for wrong level spine surgery remains problematic.

**Case Presentation:**

A 34-year-old male was referred to us to evaluate his persistent thoracic pain following right-sided microdiscectomy at T7-8 at an outside institution. Postoperative imaging showed the continued presence of a herniated disc at T7-8 and evidence of a microdiscectomy at the level immediately above. The possibility that wrong level surgery had occurred was discussed with the patient and revision surgery was planned. During surgery, the site of the previous laminectomy was clearly visualized; however, we also experienced confusion when verifying the level of the previous surgery. We ultimately used the previous laminectomy site as a landmark for identifying and treating the correct pathologic level. Postoperative consultation with Musculoskeletal Radiology revealed the patient had two abnormalities in his spinal anatomy that made intraoperative counting of levels inaccurate, including a pair of cervical ribs at C7 and the absence of a pair of thoracic ribs.

**Conclusion:**

This case highlights the importance of strict adherence to a preoperative method of vertebral labeling that focuses on the landmarks used to label a pathologic disc space, rather than simply relying on the reference to a particular level. That is, by designating the pathological level as the disc space associated with the fourth rib up from the last rib-bearing vertebrae, rather than calling it "T7-8", then the correct level can be found intraoperatively even in the case of abnormal segmentation. We recommend working closely with radiology during preoperative planning to identify unusual anatomy that may have been overlooked. We also recommend that radiology colleagues use the same system of identifying pathological levels when dictating their reports. Together, these strategies can reduce the risk of wrong level surgery and increase patient safety.

## Background

Wrong site surgery is one of five surgical "Never Events," which include performing surgery on the incorrect side or incorrect site, performing the wrong procedure, performing surgery on the wrong patient, unintended retention of a foreign object in a patient, and intraoperative/immediate postoperative death in an ASA Class I patient [1]. In the spine, wrong site surgery occurs when a procedure is performed on an unintended vertebral level. Several national protocols have been developed to decrease the incidence of wrong site surgery, such as "The Universal Protocol for Preventing Wrong Site, Wrong Procedure, and Wrong Person Surgery" from The Joint Commission and the "Sign, Mark and X-Ray" (SMaX) program from the North American Spine Society (NASS) [2,3].

Using palpable and visible anatomic landmarks alone to identify the desired vertebral level during spine surgery has been shown to be unreliable; therefore, intraoperative radiography is a critical step in identifying the correct surgical level [4-6]. However, interpreting thoracic spine imaging can be especially challenging because nearby body parts, such as the shoulder girdle and upper chest, produce radiographic shadowing over the thoracic spine. The quality of imaging is also degraded by the presence of abundant soft tissue in patients with increased BMI. Given these difficulties with interpreting imaging, intraoperative counting of vertebral levels is important in spine surgery. The sacrum and first and last rib-bearing vertebrae are typically used as landmarks for counting. However, counting from these landmarks is only appropriate if the patient has conventional numbering (i.e. 7 cervical vertebrae, 12 rib-bearing thoracic vertebrae, and 5 lumbar vertebrae) or if unconventional segmentation is clearly identified prior to surgery. If a patient has unusual vertebral column anatomy that is not recognized before surgery, then intraoperative counting can be problematic and may lead to surgery on the wrong level.

Here we report an unusual case of wrong level surgery. We discuss possible factors that led to this "Never Event" as well as recommendations for avoiding this occurrence in the future.

## Case Presentation

A 34-year-old male was referred to us from an outside institution to evaluate his persistent midback and thoracic pain in the T7 dermatome distribution. His back pain had developed approximately one year before our first encounter and an MRI performed at the outside institution showed a herniation of the T7-8 thoracic intervertebral disc. He was initially treated conservatively with non-surgical measures, including NSAIDs and epidural injections, which did not provide lasting relief. Shortly thereafter, the patient underwent a right-sided microdiscectomy at T7-8 at the outside institution; however, his symptoms did not improve. Postoperative imaging of his thoracic spine showed the continued presence of a herniated disc at the T7-8 level with evidence of a microdiscectomy at the level immediately above the herniation (T6-7). The possibility that wrong level surgery had occurred was discussed with the patient and he was referred to our institution for further evaluation and treatment.

**Figure 1:**
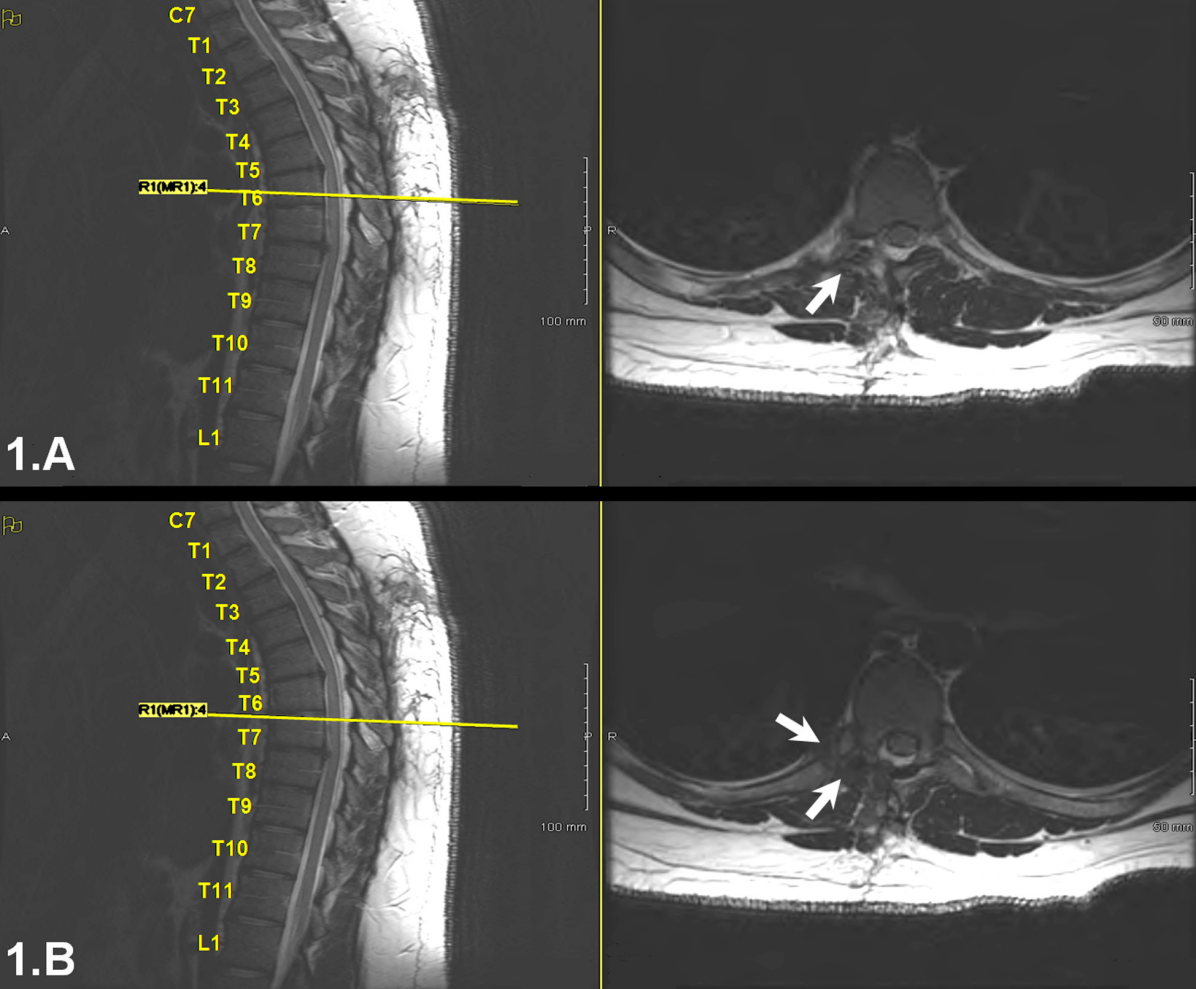
**MRI imaging after the first discectomy and decompression**. A. complete right sided laminectomy at T6-7 (arrow). B. partial laminectomy at T7, a fractured right T7 rib (double arrow).

**Figure 2:**
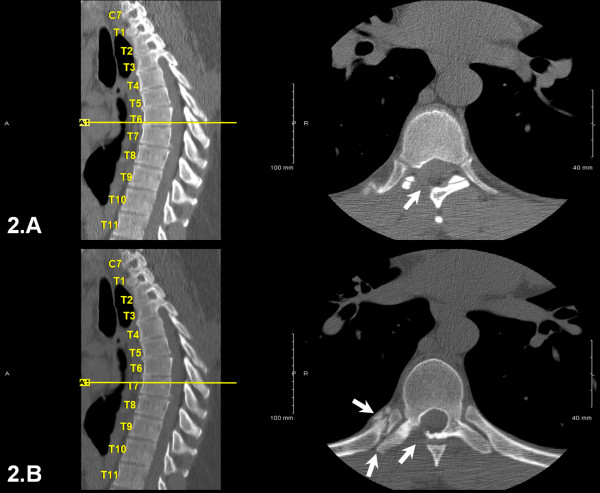
**CT scans of the thoracic spine after first discectomy and decompression**. A. complete right sided laminectomy at T6-7 (arrow). B. partial laminectomy at T7-8 (arrow), a fractured right T7 rib (double arrow).

At that time, we reviewed the patient's postoperative MRI and CT scans and agreed that the wrong level had been treated previously. We noted a hemi-laminectomy at T6 on the right side, a partial right laminectomy at T7, and a fractured right T7 rib (Figures 1, 2). Disc protrusions were visible at T7-8 and at T5-6. A right transforaminal T7-8 epidural injection was performed, which provided significant improvement within the immediate anesthetic phase. Given the success of the epidural injection, the disc herniations at T7-8 and T5-6, and in light of the rib fracture, we decided to perform a T5-T8 decompression and fusion.

## Surgery

The site of the previous laminectomy was clearly visualized during surgery. Under C-Arm guidance, two experienced spine surgeons (one neurosurgeon and one orthopedic surgeon) counted the vertebrae multiple times to verify the correct surgical levels. Counting up from both the sacrum and from the last rib-bearing thoracic vertebrae (presumably T12) to the level of the previous laminectomy resulted in that level being labeled "T7-8." In addition, counting down from the first rib-bearing vertebra (presumably T1) again resulted in "T7-8" as the level of the previous laminectomy. We then proceeded with the decompression and discectomy at the level below the previous surgery because we knew the missed pathology was immediately below the previous laminectomy. Posterior spine fusion was also performed at the levels above and below the previous laminectomy, using the previous laminectomy site as the key landmark for identifying the levels of treatment. The patient had an uneventful post-operative recovery and reported an immediate improvement from his preoperative pain.

**Figure 3:**
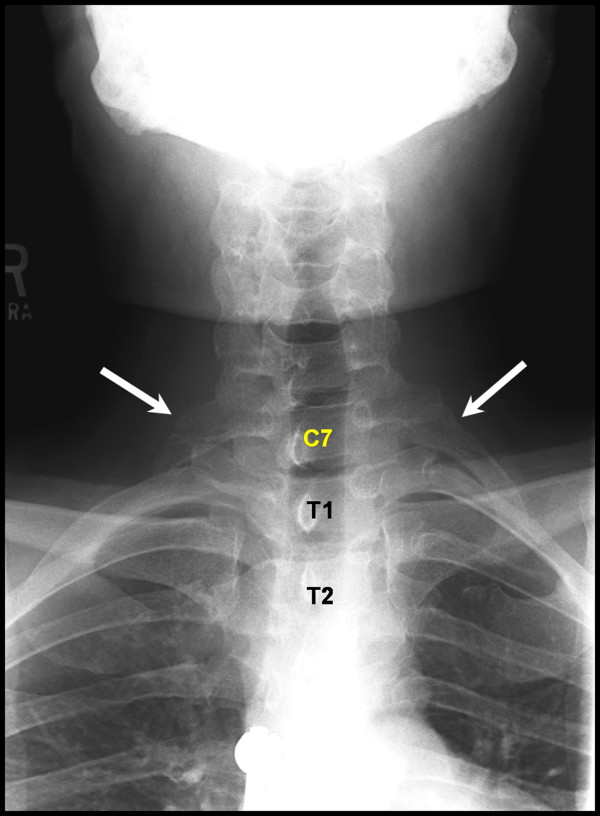
**Cervical ribs at C7**. Plain antero-posterior radiographs demonstrate C7 vertebra bearing a pair of ribs, left larger than right, which cannot be considered thoracic because they do not articulate with the manubrium.

**Figure 4:**
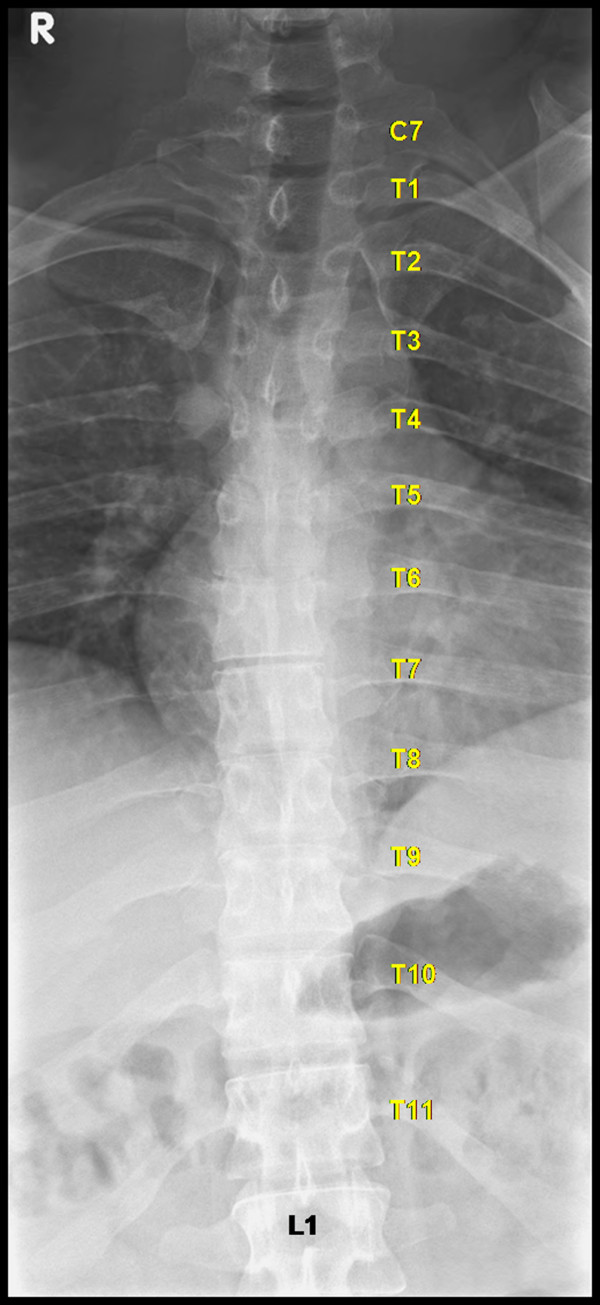
**Thoracic rib counting**. Antero-posterior radiographs of the thoracic spine demonstrate eleven pairs of true thoracic ribs.

After surgery, Musculoskeletal Radiology colleagues were consulted to evaluate our intraoperative designation of vertebral levels. Careful evaluation of the images revealed two abnormalities in the patient's vertebral segmentation that skewed our intraoperative counting of levels. First, the patient had a pair of cervical ribs at C7 (Figure 3); second, the patient had only 11 pairs of thoracic ribs (Figure 4). Thus, correct labeling of the patient's vertebrae showed that we performed posterior fusion from T5-T8 with decompression at the correct T7-8 pathologic level, even though intraoperatively we thought we were treating T6-T9.

## Discussion

In spine surgery, the first and/or last rib-bearing vertebrae are often used as a reference point to count and navigate between vertebrae. As a result, unconventional segmentation can lead to errors in identifying surgical levels. In this case report, we describe a patient who presented to us after he received wrong level thoracic surgery at an outside institution, not because the surgeons were negligent or failed to count the levels correctly, but because his spine anatomical anomalies were not recognized preoperatively. We also experienced confusion intraoperatively when attempting to verify the level of the patient's previous surgery for the same reason. We ultimately used the previous laminectomy site as a landmark for identifying and treating the correct pathologic level. After postoperative consultation with Musculoskeletal Radiology colleagues, we learned of the two abnormalities in the patient's spine anatomy, one of which included a pair of cervical ribs. Thus, when we used the first rib-bearing vertebrae to count levels intraoperatively, we mistakenly labeled that vertebra as T1, rather than C7. This resulted in us designating the site of the previous laminectomy as "T7-8," rather than T6-7. Not only did this patient have ribs at C7, but he also lacked a pair of thoracic ribs. Therefore, when we counted up from the sacrum or the last rib-bearing vertebra, we accounted for 12 thoracic vertebrae and again mislabeled the previous surgical site as "T7-8." Fortunately, we were able to use the landmark of the patient's previous wrong level surgery to determine which levels to treat intraoperatively. Based on the information available at the time of the original surgery, it is likely that we would have made the same mistake. Thus, we felt this case presented a valuable learning opportunity for the surgical community.

This case report highlights the importance of strict adherence to a method of vertebral labeling that focuses on the landmarks used to label a pathologic disc space, rather than simply relying on the reference to a particular level. That is, if the surgeon preoperatively designates the operative level as the disc space associated with the fourth rib up from the last rib-bearing vertebrae, rather than calling it "T7-8," then the correct level can be found intraoperatively even in the case of abnormal segmentation. A similar approach can be used to designate levels in the lumbar spine, particularly to prevent wrong level surgery in patients with sacralization of L5 or a vestigial disc at S1-S2. Moreover, we recommend working closely with radiology colleagues and encouraging them to use the same system of identifying pathological levels when dictating their reports. By consistently using this method to identify levels using landmarks, together with preoperative consultation with radiologists, we believe that the risk for wrong level surgery can be reduced. We now have weekly radiology conferences with our musculoskeletal radiologists to assist with preoperative planning and to identify unusual anatomy that may have been overlooked.

Despite the implementation of several national protocols to decrease the incidence of surgical 'Never Events', there is a persisting high frequency of occurrences [7]. An American Academy of Orthopaedic Surgeons task force estimated that orthopedic surgeons have a 25% chance of performing a wrong site surgery during a 35-year career, with a particularly high risk for wrong level spine fusions [8]. It is difficult to assess the true frequency of wrong level surgery in spine, since most published studies are based on self-reported answers to surveys, with response rates ranging from 12 to 68% [9,10]. In one such survey of 126 neurosurgeons, the incidence of wrong level spine surgery was estimated to be 12.8 occurrences per 10,000 lumbar discectomies and 7.6 per 10,000 cervical discectomies [9]. In another survey of 415 surgeons, 50% reported they had performed at least one wrong level surgery during their career, with an estimated prevalence of 1 in 3110 procedures [10]. However, the authors of one survey study caution that surgeons may be hesitant to report such mistakes and thus the overall frequency of wrong level surgery is likely higher [9]. The majority of wrong level procedures reported in the survey of 415 surgeons occurred in the lumbar spine (71%), followed by the cervical (21%), and thoracic spine regions (8%) [9,10]. One prospective study examining the rate of incorrect level exposure in 100 consecutive patients undergoing lumbar discectomy reported that the wrong level was initially exposed in 15% of the cases [6]. These studies clearly indicate that the risk for wrong level spine surgery remains despite the efforts of the above mentioned safety protocols.

In addition to fatigue and unusual time pressures to start or finish a procedure, the most commonly reported factors for wrong site spine surgery in one survey of surgeons were the failure to verify operative site with radiography and unusual patient anatomy [9]. A breach in standard protocol was acknowledged in 21% of the cases. Respondents in the survey also cited poor quality of imaging studies and misinterpretation of images as factors in performing surgery on the wrong level [9]. In the present case, the patient had two spine abnormalities that were not recognized preoperatively; a pair of cervical ribs and the absence of a pair of thoracic ribs. True thoracic ribs are defined as those that articulate with the manubrium. Cervical ribs either end freely in soft tissues around the neck or articulate with the first thoracic rib. Radiographic studies have reported prevalence rates of cervical ribs to range from 0.05 to 3% [11-16] and a cadaveric study of 250 cadavers (500 sides) found a 1.6% incidence of cervical ribs. Only 10% of individuals with cervical ribs will develop symptoms in the form of thoracic outlet syndrome; the majority of individuals will remain asymptomatic throughout life and only a small percentage of them will have their cervical ribs incidentally discovered [17,18]. The absence of a pair of thoracic ribs is more common, with approximately 5-8% of normal individuals having only 11 pairs of ribs [19]. In addition, it has been shown that 11 pairs of ribs are present in 33% of newborn infants with Down Syndrome [20].

To avoid the risk for wrong site spine surgery, several alternative methods of vertebral localization have been proposed in the literature. For example, Paolini et al. [21] suggest marking the vertebral spinous process preoperatively by injecting 0.3 - 0.5 ml methylene blue into the spinous process. They describe the technique as being simple and immediate and useful for preventing errors in identifying surgical targets [21]. An oblique modification of the standard cross table lateral has also been reported to improve visualization of desired thoracic levels by removing the shoulder and the majority of the ribs from the active field of view [22]. Another paper describes using preoperative percutaneous placement of polymethylmethacrylate into the vertebral body using standard vertebroplasty as an alternative for accurate localization of thoracic vertebrae [23]. Others report great success using preoperative surface localization of thoracic pathology with adhesive, disposable radiographic skin markers that are filled with radio-opaque material [24]. Although these innovative methods can likely improve intraoperative identification of thoracic spine levels, we believe that simply adhering to standard method of vertebral labeling that focuses on the landmarks used to find a pathologic disc space can reduce the risk for wrong level surgery.

## Conclusion

In this article, we describe a case in which a patient had unconventional cervical and thoracic anatomy and initially received surgery at an outside institution on the wrong thoracic level. After referral to our institution, we performed a revision surgery to treat the intended pathologic level and also experienced intraoperative confusion in determining the correct numbering of the patient's vertebrae. This case underscores the importance of strictly adhering to a method of vertebral labeling that focuses on the landmarks used to label a pathologic disc space, rather than simply relying on the reference to a particular level. We further recommend regular preoperative consultation with radiologists to identify unusual anatomy that may have been overlooked.

## Consent

Written informed consent was obtained from the patient for publication of this Case report and any accompanying images. A copy of the written consent is available for review by the Editor-in-Chief of this journal.

## Competing interests

The authors declare that they have no competing interests.

## Authors' contributions

SB and EL wrote the manuscript. EB helped to draft the manuscript. VP treated the patient, conceived the case report, and helped to draft the manuscript. All authors contributed to the revisions of the text and approved the final version of this manuscript.
